# HIV-1 sequences in the epidemic suggest an alternative pathway for the generation of the Long Terminal Repeats

**DOI:** 10.1038/s41598-017-14135-z

**Published:** 2017-10-20

**Authors:** Pierre Cappy, Alice Moisan, Fabienne De Oliveira, Jean-Christophe Plantier, Matteo Negroni

**Affiliations:** 1 Université de Strasbourg, CNRS, Architecture et Réactivité de l′ARN, UPR 9002, F-67000 Strasbourg, France; 2 Normandie Univ, UNIROUEN, EA2656 GRAM, Rouen, France; 3 CHU de Rouen. Laboratoire de virologie associé au Centre National de Référence du VIH, Rouen, F-76000, France

## Abstract

To generate the long-terminal repeats (LTR) that border the integrated viral genome, two-strand transfer steps must occur during reverse transcription. Analysis of the genetic polymorphisms that are present in the LTR of HIV-1 heterozygous virions in single infection cycle studies has revealed which of the two copies of genomic RNAs is used for each transfer event. Thus, the first event of strand transfer has been described to be either intra- or intermolecular, while the second event is generally intramolecular. Here, we repeated these analyses using sequences from HIV databases and extended the study to the regions surrounding the LTR. We observed a striking correlation between the pattern of recombination in the LTR and the phylogenetic origin of the surrounding sequences. This correlation suggests that the second-strand transfer can be either intra- or intermolecular and, interestingly, could reflect an effect of proximity between nucleic acids that would guide this transfer. This factor could be particularly relevant for heterozygous viruses containing highly divergent genomic RNAs, such as those considered in the present study.

## Introduction

**Figure 1:**
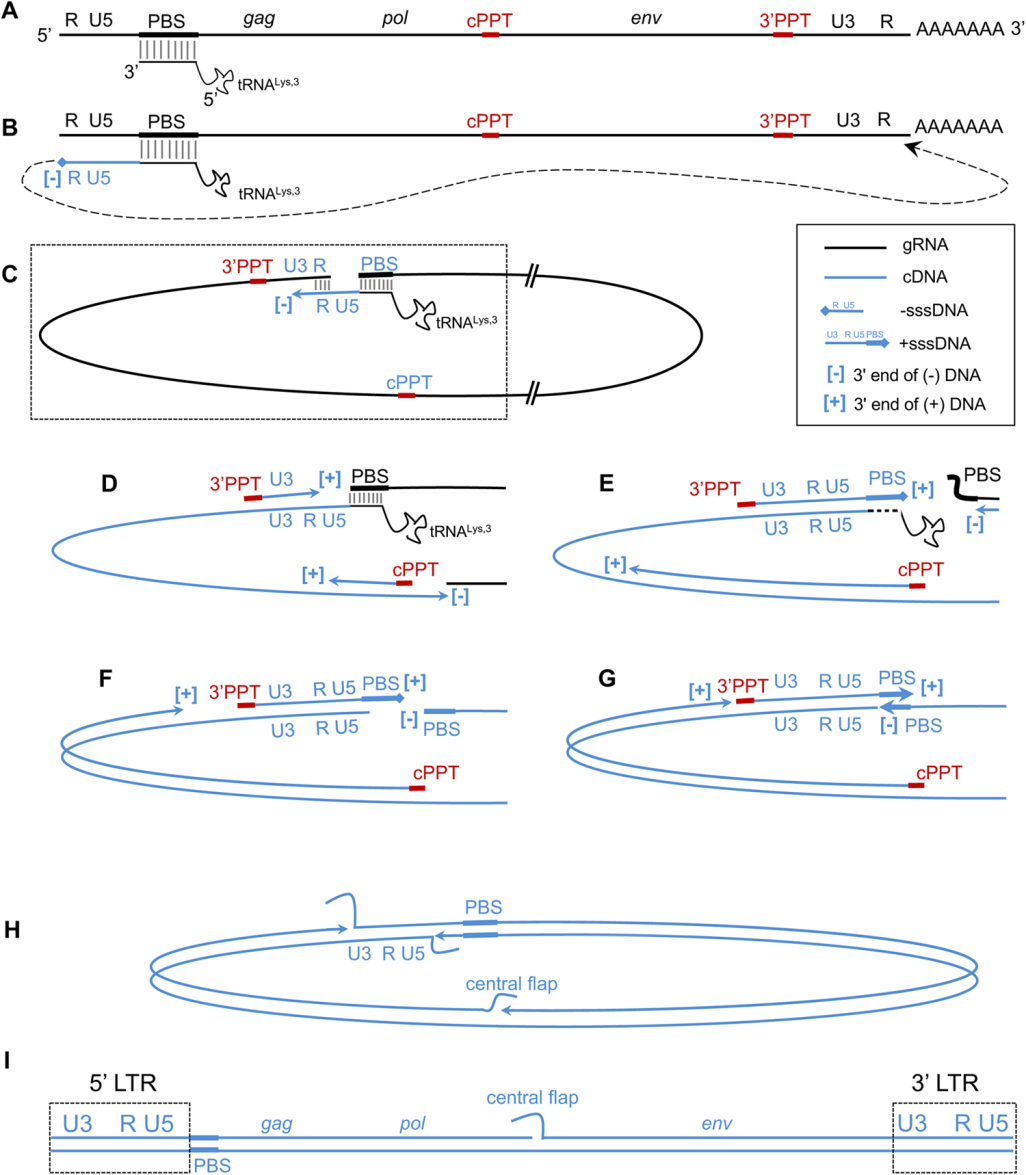
Process of reverse transcription in HIV-1. Panel A, schematic representation of the gRNA with the main genes and the elements constituting the LTR (R, U5 and U3). RNA is drawn in black, DNA in blue. The PBS sequence is indicated by a thicker box, black for RNA and blue for DNA. The central and the 3′PPT RNA sequences are indicated by thicker red boxes. The tRNA^Lys3^ used to prime reverse transcription is drawn annealed to the PBS (the extent of the annealed regions throughout the picture is not in scale). Panel B, Minus DNA synthesis is primed by the tRNA and reaches the 5′ end of the gRNA before first strand transfer occurs (dotted arrow) due to the presence of the repeated sequence R. Panel C, this leads to the circularisation of the gRNA and allows the resumption of DNA synthesis. The boxed region is the one that will be drawn in panels D-G. Panel D, synthesis of the (−) DNA strand proceeds generating the first LTR sequence and continues through the internal regions of the genome. 3′PPT and cPPT are resistant to the RNase H cleavage by the RT and prime synthesis of the (+) DNA strand. Panel E, (+) strand DNA synthesis started from the 3′PPT reaches the tRNA^Lys,3^, copies the 18 nt at its 3′ end and removes the remainder of the tRNA. This generates + sssDNA and displaces the PBS portion of the gRNA. Panel F, (−) DNA synthesis copies the PBS sequence of the gRNA and degrades it. Panel G, the complementary PBS sequences anneal (second strand transfer) allowing the DNA synthesis to resume. Panel H, (−) DNA strand synthesis displaces the (−) DNA LTR strand ahead (that serves as template for (+) DNA strand) generating one double stranded 5′ LTR. (+) DNA synthesis from the cPPT displaces the (+) DNA strand generated from the 3′PPT and generates the double stranded 3′ LTR. (+) DNA synthesis initiated from the 3′PPT reaches the (+) DNA strand primed by the cPPT and only partially displaces it before stopping definitively at the central termination site, generating the central flap. Panel I, structure of the complete pre-proviral DNA; the LTR sequences are boxed.

The replication of the retroviral genome requires the conversion of the viral single-stranded genomic RNA to double-stranded DNA by the viral reverse transcriptase (RT)^1,2^. To achieve this goal, the nascent DNA strands must be transferred at least twice from one region of the template onto another, as schematized in Fig. 1A–I. In particular, two events, named first and second strong stop strand transfer (hereafter referred simply as first and second strand transfer, respectively), are required to achieve the synthesis of a complete proviral DNA molecule with the duplicated long terminal repeated sequences (LTR) that constitute the viral promoter. Additional strand transfer events between the two copies of genomic RNA (gRNA) contained in a viral particle can occur during copying of internal regions of the genomic RNA (copy choice)^3^. When the two RNAs carry distinct genetic polymorphisms, these strand transfer events can lead to genetic recombination.

The mechanism underlying the generation of LTR has been intensively studied in different retroviruses by exploiting the genetic polymorphisms between the two RNA copies in heterozygous virions to determine which gRNA was used for each event of strand transfer^4–6^. For the human immunodeficiency virus (HIV), studies of a single infection cycle in cell culture have concluded that the first strand transfer can be intra- or intermolecular with comparable frequencies. In the first case, minus-strand strong-stop DNA (-sssDNA) is transferred to the 3′ end of the same gRNA used as the template for its synthesis, while in the second event, -sssDNA is transferred onto the 3′ end of the second copy of gRNA present in the virion. By contrast, the second strand transfer is generally intramolecular (see panels F and G in Fig. 1), indicating that the 5′ ends of the annealing (−) strand DNA and (+) strand DNA PBS sequences originate from the same gRNA strand rather than from an inter-strand interaction.

To address the question of the relative use of the two gRNAs for the generation of the LTR using sequences issued from natural infections, one must rely on the sequences of recombinant viruses available in databases. The region where DNA synthesis switches between the two genomic RNAs, generating the recombinant DNA is defined as a breakpoint. Specifically, a breakpoint corresponds to the region encompassed by the two closest informative sites of each of the parental strains involved in the crossing. As such, its size varies as a function of the density of these sites, with one nucleotide being the smallest size and without an actual upper size limit. In HIV databases, while breakpoints have been mapped along the coding sequences, little is known about the breakpoints in the LTR. However, the increasing numbers of available sequences now also enable the characterization of recombinant LTR from patient isolated strains^7–14^.

A plethora of HIV recombinants have been identified in the last few decades^15,16^. HIV is subdivided into two types, HIV-1 and HIV-2. No natural recombinants between these two types have been described so far. HIV-1, the type responsible for the AIDS pandemic, is divided into four groups: M, N, O, and P. Inter-group recombinants have been described between groups M and O *in vivo* ^7–9,14,17,18^. Within groups, recombinants have largely been documented for group M^16,19^ and to a lesser extent for group O^20^. Recombinants found in fewer than three patients are called unique recombinant forms (URFs), while those found in at least three unrelated patients are called circulating recombinant forms (CRFs)^21^. The present work is based on the analysis of all three classes of recombinants identified so far in natural infections: intra-group M CRFs and URFs, and MO inter-group recombinants (HIV-1/MO). We exploited the sequence divergence that exists *in vivo* in chimeric genomes with recombinant LTR to verify whether the signature of their mechanisms of generation is consistent with that expected based on the commonly accepted models. To this end, we extended the analysis of the pattern of recombination not only to the LTR but also to the surrounding sequences.

## Results

### Analysis of the available CRFs, URFs and HIV-1/MO sequences

**Figure 2:**
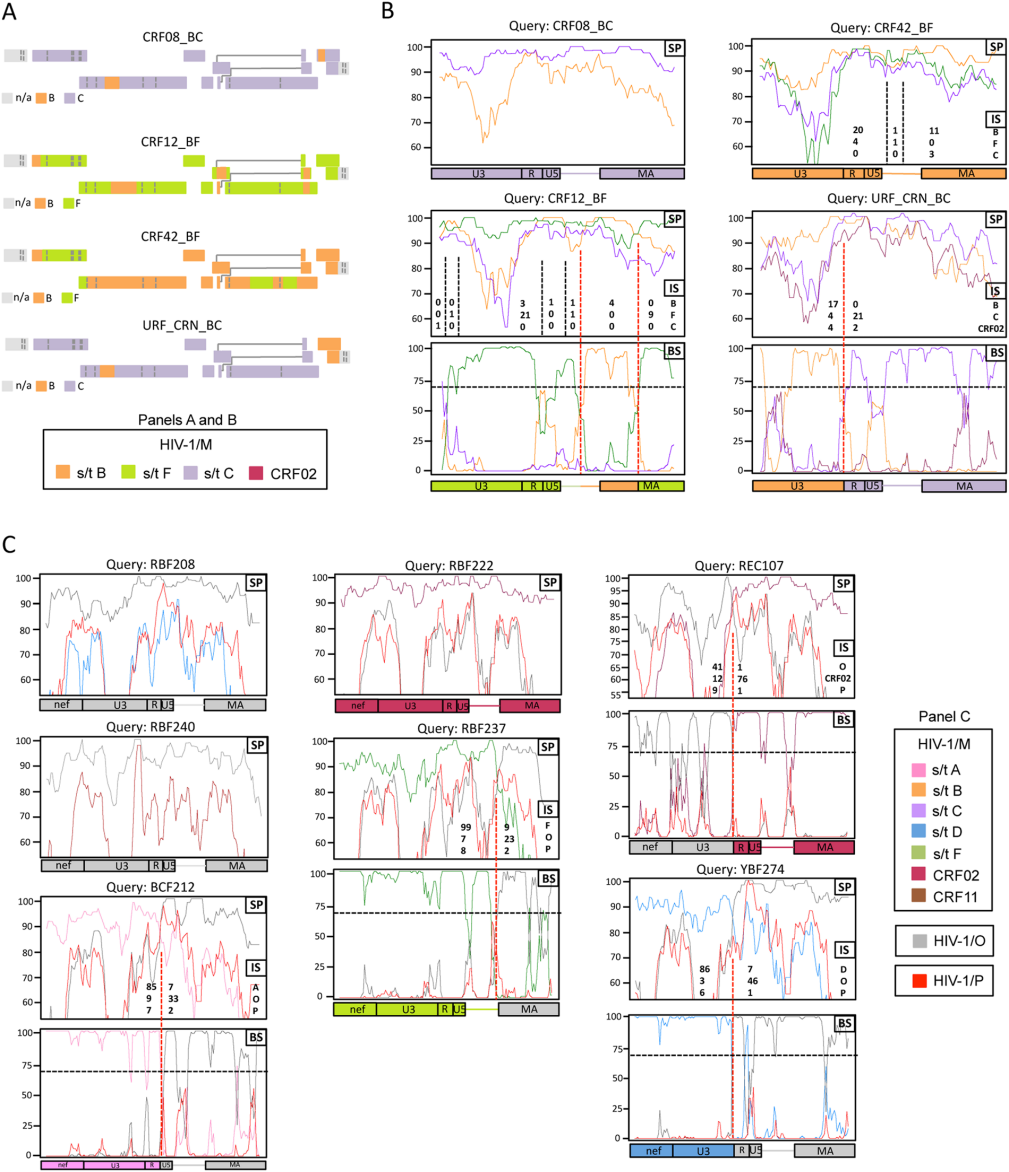
Study of the pattern of recombination for the samples for which a detailed analysis of the LTR-MA or nef-LTR-MA regions was not available. Panels A and B, Analysis of CRF08, CRF12, CRF42 and URF_CRN in the LTR-MA region. The same colour code is used for HIV-1/M subtypes (s/t) in the two panels. Panel A, Recombination pattern obtained from the analysis of the sequences using jpHMM. Boxes represent HIV ORFs, coloured according to the subtypes. jpHMM does not analyse the non-coding region of the LTR (n/a: not applicable). Panel B, recombination analysis for intra-M recombinants in the LTR-MA region, computed with SimPlot. Similarity plots (SP) were first drawn for each strain (the y-axis represents the percentage of similarity between strains). The number of informative sites (IS) corresponding to each subtype (given on the right of the panels) are shown for each interval separated by putative cross-overs (vertical dashed-lines). When the Yates-corrected X^2^ test yields a p-value < 0.01, the distribution from each side of the line was considered to be significantly different, and the line is shown in red, defining the position of the breakpoint. BootScan (BS) analyses were then carried out to confirm the breakpoints location (the y-axis represents the percentage of permuted trees and the 70% threshold is indicated by the horizontal dashed line). Panel C, Analysis of HIV-1/MO recombinants RBF208, RBF222, REC107, BCF212, YBF274, RBF237 and RBF240 in the *nef*-LTR-MA concatenated region. The analysis is the same as in panel B.

Starting with the sequences available in databases, we exploited the sequence diversity found *in vivo* in heterozygous virions to investigate which of the two copies of genomic RNAs is used during first and second strand transfers (Fig. 1B,C and F,G, respectively). This approach requires focusing on recombinants, as these viruses are necessarily generated from heterozygous virions. Because, as mentioned above, we wanted to extend the analysis of the recombination pattern not only to the LTR but also to the surrounding sequences, we decided to exclusively consider the recombinant forms for which the full-length sequence, including the LTR, was available (see Materials and Methods). For the sequences of HIV-1/M CRFs and URFs, data on the complete recombination pattern of 13 HIV-1/M CRFs (subtype G^22^, CRF03_AB, CRF08_BC, CRF12_BF, CRF26_AU, CRF31_BC, CRF32_06/A1, CRF42_BF, CRF45_cpx, CRF60_BC, CRF71_BF, CRF87_cpx, CRF88_BC) and that of 5 URFs (URF_SHI^10^, URF_CRN^11^, URF_AUK^11^, URF_WEI^13^, URF_GUI^12^) were available. Concerning CRF08, CRF12, CRF42 and URF_CRN, the precise characterization of the recombination pattern in the region encompassing the LTR and the matrix-coding sequence (LTR-MA) was missing from the databases. We therefore completed the recombination analysis in this region using SimPlot^23^ (Fig. 2A,B). For MO recombinants, nine fully sequenced recombinants were considered (see Materials and Methods). For seven of these recombinants (RBF208^8^, YBF274, REC107, BCF212, RBF222, RBF237 and RBF240), the precise characterization of the recombination pattern in *nef* and the LTR-MA region was also lacking. These recombinants were therefore reanalysed here (Fig. 2C). Altogether, 27 full-length recombinant sequences were considered, including 13 CRFs, 5 URFs and 9 HIV-1 intergroup recombinants from groups M and O (Supplementary Table S1).

### Pattern of recombination in fully sequenced CRFs, URFs and HIV-1/MO recombinants

In heterozygous viruses, the synthesis of the LTR results in well-defined patterns of recombination. Intermolecular first strand transfer generates recombinant LTR with a breakpoint at the border between U3 and R (U3/R junction), while intramolecular first strand transfer leads to LTR without breakpoints. For second strand transfer, which occurs in the primer binding sequence (PBS), intermolecular transfer creates a breakpoint between U5 and the 5′ portion of *gag* (U5/*gag* junction). Intramolecular second strand transfer, instead, generates LTR without breakpoints. For the analysis of the generation of the LTR we therefore focused on the breakpoints located at the U3/R and U5/*gag* junctions. To obtain a more comprehensive view of the dynamics underlying the use of the two gRNAs during the generation of the LTR, we also analysed the pattern of recombination in the rest of the genome, focusing on the phylogenetic origins of the regions adjacent to the LTR: the *nef* and *gag* genes. These sequences are indicative, respectively, of which RNA was used for the first strand transfer and on which RNA (−) DNA synthesis was achieved before the second strand transfer.

**Figure 3:**
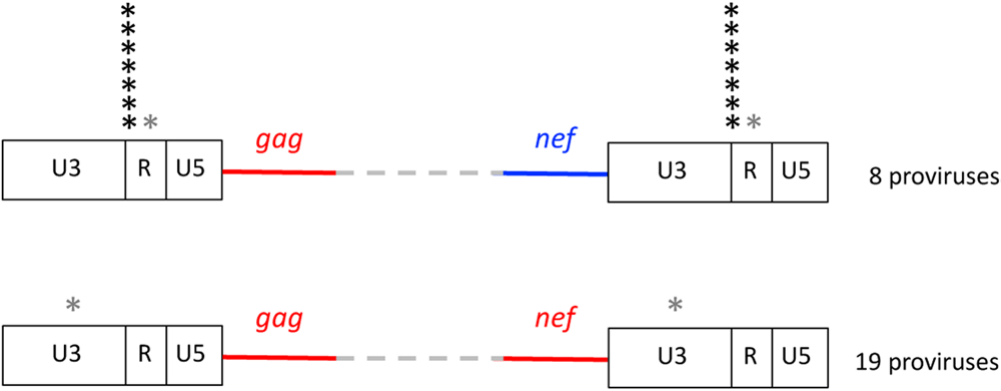
Localisation and number breakpoints found in the LTR. The asterisks indicate the location of individual breakpoints identified with our analyses. The breakpoints attributed to copy choice are shown in grey.

We observed a striking trend when the proviruses were divided into those presenting phylogenetically matching *gag* and *nef* (*“matching gag-nef”* proviruses, 12 CRFs, 3 URFs and 4 HIV-1/MO) and those in which these sequences are phylogenetically discordant (*“discordant gag-nef”* proviruses, 1 CRF, 2 URFs and 5 HIV-1/MO). Indeed, the pattern of the breakpoints in the LTR markedly differs between the two classes (Table 1 and Fig. 3). Among 19 *matching gag-nef* recombinants, only one recombinant presented a breakpoint in the LTR, and its location is indicative of generation by copy choice within U3 (Fig. 3). By sharp contrast, 8/8 of the *discordant gag-nef* proviruses presented breakpoints in the LTR. Specifically, except for one breakpoint the pattern of which suggests its generation by copy choice within the R region, the other 7 breakpoints were located at the U3/R junction (Fig. 3). This distribution is significantly unequal (p < 10^−4^), suggesting a link between the positions of the breakpoints in the LTR and the phylogenetic origin of their surrounding sequences. As the phylogenetic origin of the different portions of the proviral DNA is indicative of which gRNA was used for reverse transcription in heterozygous virions, we discuss below how this observation can be exploited to determine whether a mechanistic relationship exists between the LTR and the surrounding regions.

**Table 1:** Classification of breakpoints in the LTR according to the presence of *matching* or *discordant gag-nef* sequences.

		**CRFs**	**URFs**	**HIV-1/MO**	**Total**
matching *gag-nef* (II)	no bkpt in LTRs	11	3	4	18
	U3/R bkpt	0	0	0	0
	U5/*gag* bkpt	0	0	0	0
	copy choice bkpt	1	0	0	1
	total II	12	3	4	19
discordant *gag-nef* (I)	no bkpt in LTR	0	0	0	0
	U3/R bkpt	1	2	4	7
	U5/*gag* bkpt	0	0	0	0
	copy choice bkpt	0	0	1	1
	total I	1	2	5	8
	total I + II	13	5	9	27

## Discussion

The generation of retroviral LTR requires two strand transfer events, called first and second strand transfers. These events can be either intra- or intermolecular between the two copies of gRNA present in the viral particle. Inter and intramolecular transfers generate precise patterns of recombination in heterozygous virions. Proviruses with a recombination breakpoint at the U3/R junction are generally attributed to intermolecular first strand transfer, while intramolecular first strand transfer yields LTR without breakpoints. As intra- and intermolecular first strand transfer occur at comparable rates in cell culture^5,6^, both types of LTR (with a U3/R breakpoint and without breakpoints) are expected to be identified in databases. Although we consistently detect both types of LTR, their distribution is unexpectedly unequal when the proviruses are grouped according to the phylogenetic origin of the sequences adjacent to the LTR (*gag* and *nef*). Indeed, LTR containing a U3/R breakpoint are exclusive to proviruses with phylogenetically *discordant gag-nef* sequences, while all LTR without breakpoints are observed among proviruses with phylogenetically *matching gag-nef*. How can this phenomenon be explained if the first strand transfer follows an intra- or intermolecular pathway on a stochastic basis?

One potential explanation is selection occurring on the recombinant LTR. As the present analysis was conducted on sequences retrieved from patients, the recombinants most likely emerged from selection for the most adapted forms *in vivo*. It is therefore possible that selective pressure erases all signatures of the mechanism of generation of the recombinant LTR. However, this explanation suggests that the presence of a breakpoint at the U3/R junction favours viruses with discordant *gag* and *nef* sequences. Intermolecular first strand transfer, generating U3/R breakpoints, would uncouple the TAR element in R from the Tat coding sequence located near the 3′ end of the genome. As the function of Tat is exerted through binding to TAR, the phylogenetic concordance could influence viral fitness; nevertheless, this factor is unrelated to the concordance or discordance of *gag* and *nef*. Consistently, the phylogenetic concordance of the Tat/TAR pair was independent of the phylogenetic concordance of the *gag*-*nef* pair (Supplementary Table S2). Another possibility, based on selection, could be that the recruitment of Gag to the plasma membrane is increased when Nef and Gag are phylogenetically concordant. Although Nef improves Gag intracellular trafficking, this effect is not exerted through a direct interaction between the two proteins^24^. Therefore, the phenomenon should not be influenced by the phylogenetic concordance between *gag* and *nef*.

Another possible explanation is that the bias is generated through the mechanism of the first strand transfer. This would imply that intermolecular transfer favours the subsequent occurrence of an odd number of internal template switching, while intramolecular transfer leads to either no or an even number of internal template switching (Supplementary Figure S1). However, although intermolecular first strand transfer increases the frequency of internal template switching by negative interference^25^, no correlation between an odd or an even number of strand transfer events and the nature of the first strand transfer has been reported.

**Figure 4:**
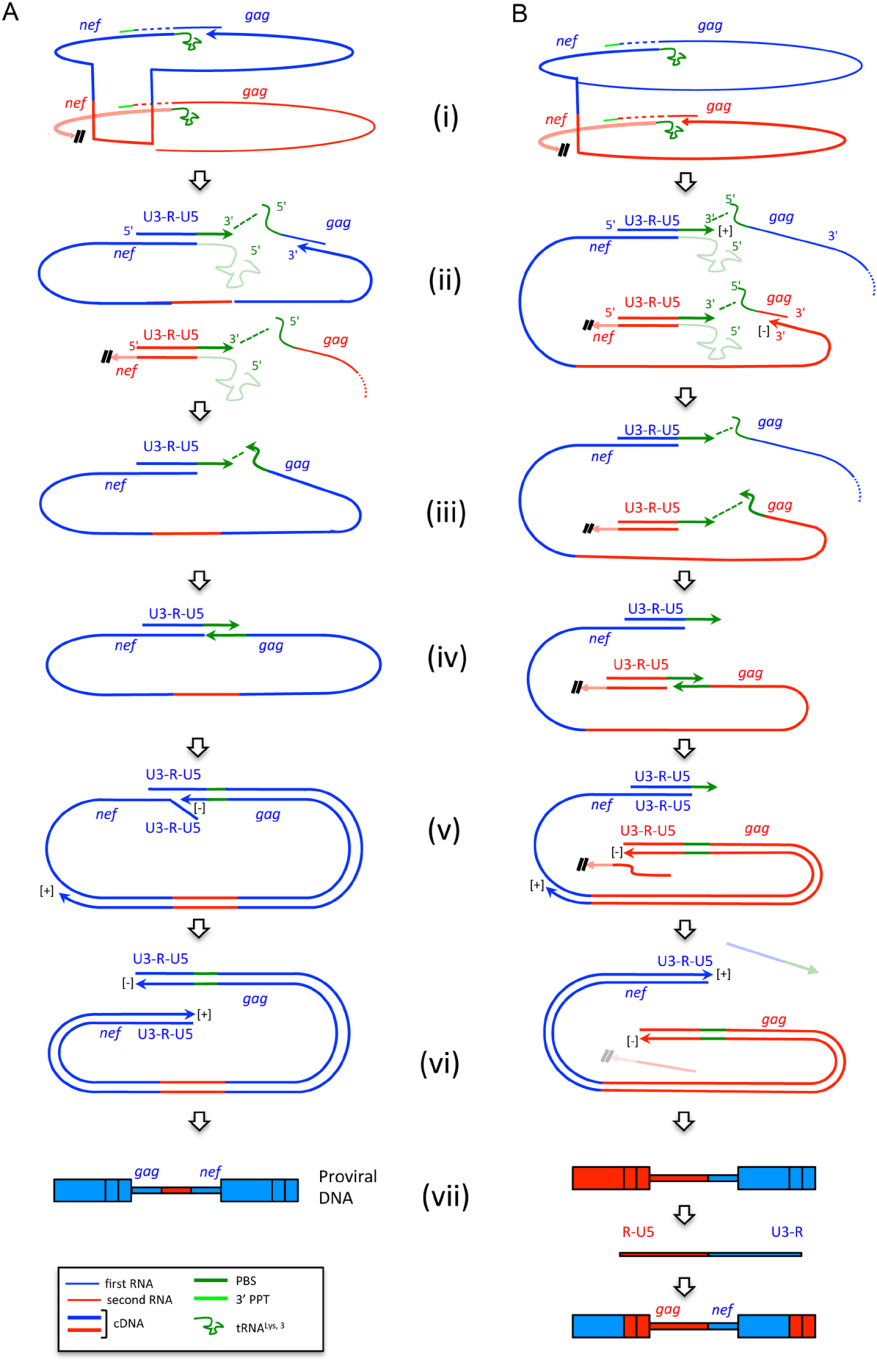
Pathways by which the first strand transfer could generate proviruses with non-recombinant LTR and *matching gag-nef* pairs (panel A) or proviruses with LTR having a breakpoint at the U3/R junction and *discordant gag-nef* pairs (panel B). Arrows indicate 5′ to 3′ polymerisation of DNA. For clarity, synthesis of the (+) DNA strand from the central PPT is omitted. (i) After priming of reverse transcription on one RNA (blue), the transfer of synthesis blocks irreversibly (double slash) a potential ongoing reverse transcription of the second RNA (in red). (ii) Minus DNA synthesis proceeds toward the 5′ end of the gRNA. + sssDNA is synthesised on both RNAs and the tRNAs (shaded) are removed. The 5′ end of the RNAs and the corresponding + sssDNA are kept in proximity (green dotted line). (iii) Minus DNA synthesis is completed by synthesizing the (−) PBS DNA (green). Molecules no longer involved in the next steps have been omitted. (iv) Second strand transfer takes place, guided by the effect of proximity indicated by the dotted green line at step iii. (v) DNA synthesis is resumed after second strand transfer and displaces the pre-existing strands of the LTR. (vi) Synthesis of the full-length pre-proviral DNA is achieved (the strands displaced in panel B are lost – shaded –). (vii) The structure of the resulting proviral DNAs is shown. The proviral DNA in panel B will generate a breakpoint at the junction U3/R at the next generation.

**Figure 5:**
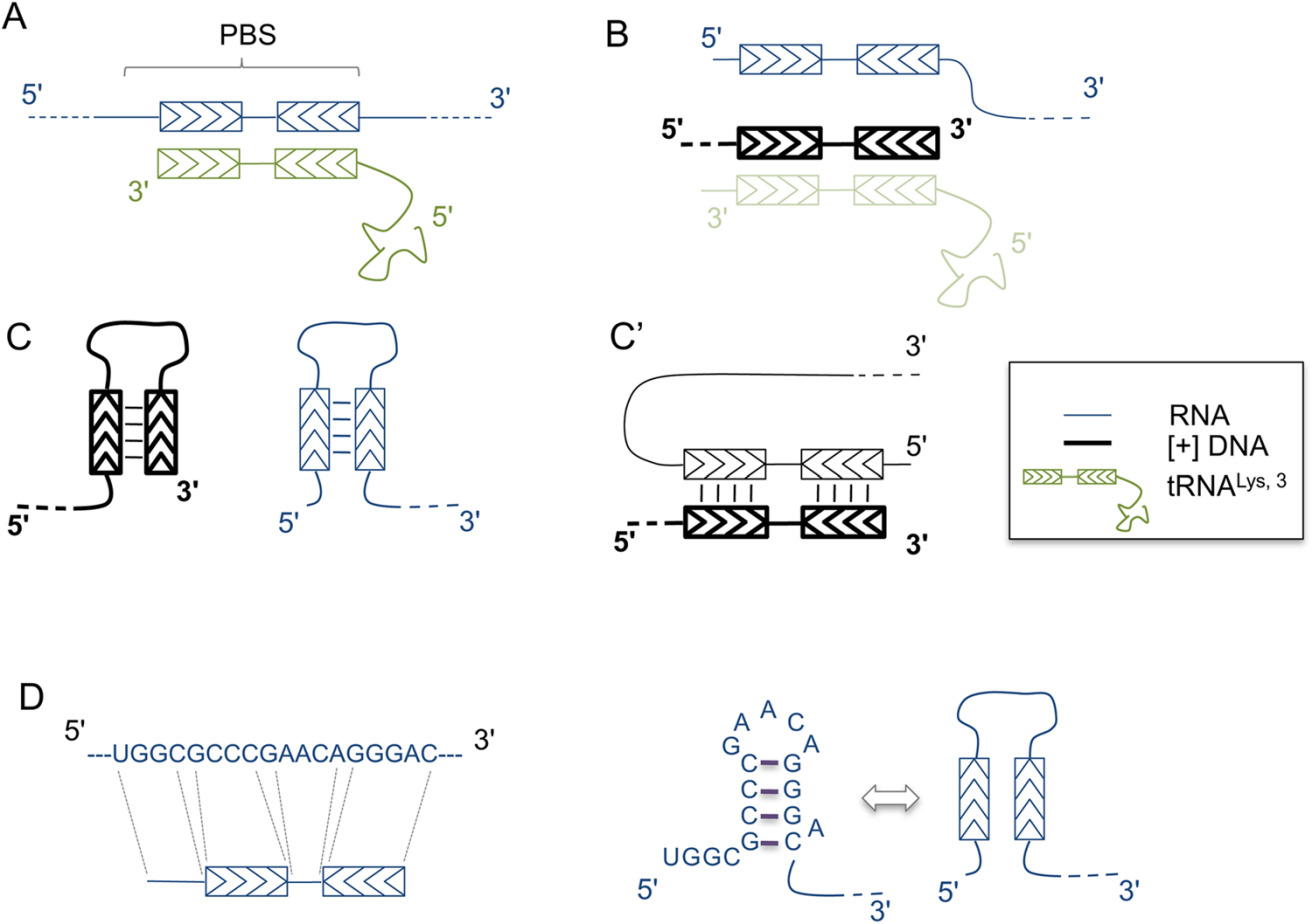
Model for how palindromic sequences in the PBS could promote spatial proximity between the genomic RNA displaced during the synthesis of + sssDNA and the + sssDNA itself. In the scheme, regions that harbour chevrons in the opposite orientation (inverted repeats) can anneal. Panel A, PBS annealed to the tRNA 3′ end (genomic RNA in blue, tRNA in green). Panel B, formation of + sssDNA after copying the cPBS component of the tRNA, displacement of the genomic RNA and the degradation of the tRNA (indicated by shading). Once the tRNA degraded, the (+) DNA PBS and the genomic RNA PBS can either form intramolecular hairpins (panel C) or a RNA/DNA heteroduplex (panel C’). Panel D, RNA sequence of the PBS (isolate MAL) with indicated the palindromic sequences involved in the formation of the stem of a hairpin according to Isel and colleagues^26^, given as an example for the possible presence of palindromic sequences in the PBS.

Alternatively, the biased presence of U3/R breakpoints could reflect the fact that these breakpoints are not generated during first strand transfer but are a consequence of the second strand transfer. In this case, the unequal distribution would result from an effect of proximity between the 5′ end of the genomic RNA displaced by the synthesis of + sssDNA and the + sssDNA itself (see panels E-G of Fig. 1). This proximity would guide the second strand transfer and, if *gag* is copied into (−) DNA using the same RNA on which (-) DNA synthesis was primed (Fig. 4A, step i), the second strand transfer would preferentially be intramolecular (Fig. 4A, steps ii-iv). This generates LTR without breakpoints and *matching gag-nef* proviruses (Fig. 4A, steps v-vii). If, instead, copying of *gag* is completed on the other gRNA (Fig. 4B, step i), the effect of proximity would favour intermolecular second strand transfer (Fig. 4B, steps ii-iv), resulting in *discordant gag-nef* proviruses that, at the next infectious cycle, would generate a breakpoint at the U3/R border (Fig. 4B, steps v-vii). U3/R breakpoints and *discordant gag-nef* proviruses would thus be associated. Palindromic sequences in the HIV-1 PBS, which have been shown to fold into small hairpins^26,27^, could favour the proximity of the 5′ end of the genomic RNA displaced by the synthesis of + sssDNA and the + sssDNA itself, as schematically indicated in Fig. 5.

Several factors may be responsible for the discrepancies between the present observations and the expectations from mechanistic studies in cell culture. By attributing the generation of the U3/R breakpoints to the second strand transfer in the analysed proviruses, we indirectly implied that in these cases, the first strand transfer is intramolecular. The genetic divergence between the sequences considered here could be a major reason for this observation. As intermolecular first strand transfer is disfavoured by sequence divergence in R^28,29^, which is higher between the reference sequences used in the present study (5.7% intra M, 3.9% intra O and 24.9% between M and O) than between the isolates used in cell culture experiments (1%^5,6^), this could explain the discrepancy between the observations published previously and ours.

The likelihood that the U3/R breakpoints identified here are generated through intermolecular second strand transfer, in contrast to the general model, is also supported by the following consideration. Depending on which RNA (−) DNA synthesis is achieved, intramolecular second strand transfer should generate, or not, a breakpoint at the U5/*gag* junction (Supplementary Figure S2). Since, due to frequent recombination in internal regions of the genome, (−) DNA synthesis should be stochastically achieved on one gRNA or on the other, breakpoints at the U5/*gag* junction should also occur on a stochastic basis, if the second strand transfer is always intramolecular. However, no U5/*gag* breakpoints were identified among the 27 analysed proviruses. Nevertheless, this difference between the observations made in the present study and those made in previous studies in single infection cycle systems could partly reflect a disequilibrium of the RT initiation on both gRNAs. Indeed, for intermolecular second strand transfer to occur, reverse transcription must begin on both types of gRNAs and + sssDNA must be correctly generated on the second gRNA. Yu and colleagues^5^ showed that the initiation of reverse transcription was three times less frequent on the second gRNA, potentially precluding the frequent generation of ( + sssDNA on this molecule. How frequently + sssDNA is generated on both gRNAs *in vivo* is unknown, but this factor likely influences the nature of the second strand transfer.

In conclusion, the results of the present study underscore an unexpected correlation between two features of the sequence of recombinant proviral DNA identified in patients, suggesting that the choice of the templates used for the generation of the LTR could also follow alternative pathways with respect to the well-established pathways. These alternative pathways could be relevant when considering heterozygous virions involving considerably divergent phylogenetic strains *in vivo*.

## Materials and Methods

### Samples

Eleven full-length CRF sequences were retrieved from the Los Alamos HIV sequence database (www.hiv.lanl.gov/). Five full-length URF sequences were searched in the GenBank with the key words “HIV URF” or “HIV recombinant full sequence”, and the 200 first hits were screened. Lots of sequences were full-length in the coding region but not in the LTRs and were therefore discarded. Eight full-length sequence HIV-1/MO recombinants were retrieved from the literature (97CA-MP645^7^, RBF208^8^, REC024^9^, YBF274^14^) or from the database of the virology unit at Rouen University Hospital (REC107, BCF212, RBF222 and RBF237; sequences in process of publication). Strains that were already described in publications and in LANL were not further analysed. All the strains used in this study are presented in Supplementary Table S1.

### Reference panel

To study the phylogenetic relationships and the recombination patterns of the HIV-1/MO intergroup recombinants as well as the HIV-1/M intragroup recombinants (CRFs and URFs), a panel of HIV-1/M reference strains was generated with sequences from HIV-1/M pure subtypes, CRF01_AE and CRF02_AG, as well as HIV-1/O and HIV-1/P sequences, gathered from the LANL sequence database and aligned with MEGA 7^30^. In this study, we considered that subtype G is a recombinant and CRF02 a pure subtype, as demonstrated by Abecasis *et al*. in 2007^22^. Very few sequences were complete in the LTRs. Namely no sub-subtype A2 and F2, and no subtypes H, J and K were available (Supplementary Table S3).

### Study of the mosaic structure of the HIV recombinants

Three CRFs (CRF08, CRF12, CRF42) from the LANL HIV database were not fully described in the LTR. One URF (URF_CRN) was fully described but one breakpoint, located between U5 and the beginning of *gag* was not precisely defined. We thus retrieved the full sequence of these four viruses for further analysis.

The coding sequence of these four strains was computed with jpHMM software^31^ (http://jphmm.gobics.de) to generate and verify the mosaic structure in their coding sequence (Fig. 2A). Moreover, for each of the four strains, the sequence going from the 5′ border of the LTR till the end of the MA coding sequence (LTR-MA) was aligned with the panel of selected HIV-1/M reference strains to study the precise recombination pattern in this region. The alignment was gap-stripped and the SimPlot software^23^ was used to perform similarity plot (SP), informative sites (IS) and BootScan (BS) analyses (Fig. 2B). For each recombinant, the SP analysis was performed using HIV-1/M subtype reference sequences corresponding to the strains whose sequences were present in the coding region of the recombinants (for instance, subtype B and C for CRF08_BC), with parameters set to 80-nt window/10-nt steps and a Kimura 2-parameter method with a transition-transversion (Ts/Tv) ratio of 2.0. For informative sites (IS) and BootScan (BS) analysis, a third HIV-1/M subtype was taken as out-group. The IS analysis was run when a cross-over was suspected using SP and consisted in determining the number of signature polymorphisms for each subtype involved in the cross-over. The distribution of the informative sites flanking the cross-over was then tested for statistical significance sites using the Yates-corrected X^2^ test, as already described^32,33^. The BS analysis was then run to confirm the location of the breakpoint.

Concerning HIV-1/MO recombinants, the non-coding sequence of the RBF208 HIV-1/MO recombinant^8^ was not described and HIV-1/MO recombinants (YBF274, REC107, BCF212, RBF222, RBF240 and RBF237) are not yet published. For these recombinants, the *nef* coding sequence and the LTR-MA region were analysed. For each recombinant, the part of the *nef* coding sequence that does not overlap with U3 was concatenated with the LTR-MA sequence into a *nef-LTR-MA* sequence (see Supplementary sequences). The *nef*-LTR-MA sequences were aligned with the reference panel with MEGA 7^30^, the alignment was gap-stripped and a recombination analysis was performed with the SimPlot software^23^ as described for HIV-1/M CRFs and URFs. For each recombinant, the SimPlot analysis was performed using HIV-1/O reference sequences and the HIV-1/M subtype reference sequences corresponding to the strain whose sequence was present in the coding region of the recombinants (for instance, subtype D for RBF208, which is a recombinant between HIV-1/O and HIV-1/M subtype D). For informative sites and BootScan analysis, HIV-1/P was taken as out-group.

### Statistical analyses

Statistical analyses were carried out with Prism 6 software. The Yates-corrected chi-square test was used to determine whether significant evidence of recombination existed using informative site (α = 0.01). The Fisher’s exact test was used to compare the distribution of the LTR breakpoints between the *matching gag-nef* and *discordant gag-nef* categories and the association of phylogenetic matching Tat and TAR as function of the concordance of *gag* and *nef* (α = 0.01, two-tailed p-value).

## Electronic supplementary material

Supplementary information
