# Supplementary material for: HIV-1 sequences in the epidemic suggest an alternative pathway for the generation of the Long Terminal Repeats
Source: Sci Rep. 2017 Oct 20;7:13715. doi: 10.1038/s41598-017-14135-z (PMC5651808; doi:10.1038/s41598-017-14135-z)
Supplement: Supplementary file 1 — Supplementary information [file 41598_2017_14135_MOESM1_ESM.pdf]

HIV-1 sequences in the epidemic suggest an alternative pathway for the generation of the Long Terminal Repeats

Pierre Cappy[1,2,3], Alice Moisan[2,3], Fabienne De Oliveira[2], Jean-Christophe Plantier[2,3] and Matteo Negroni[1*]

[1]Université de Strasbourg, CNRS, Architecture et Réactivité de l'ARN, UPR 9002, F-67000 Strasbourg, France; [2]Laboratoire de Virologie, Associé au Centre National de Référence du VIH, Hôpital Charles Nicolle, CHU de Rouen, Rouen, France; [3]GRAM EA2656, Faculté de Médecine-Pharmacie, Normandie Université, Rouen, France

Supplementary information

Table S1. Recombinant strains retrieved and used in the study (HIV-1/MO, HIV-1/M CRFS and URFS).

Identification	Type of recombinant	Reference	GenBank accession number
97CA-MP645	HIV-1/MO	Peeter et al., 1999, J virol	AJ239083
REC024	HIV-1/MO	Ngoupo et al., 2016, AIDS	KM438032
RBF208	HIV-1/MO	Vessière et al., 2010, AIDS	GQ351296
YBF274	HIV-1/MO	De Oliveira et al., 2017, Retrovirology	KX579838
REC107	HIV-1/MO	Ngoupo et al., 2016, 21th Int. AIDS Conf., 2016, Durban	N/A
BCF212	HIV-1/MO	N/A	KY359380
RBF222	HIV-1/MO	N/A	KY359382
RBF237	HIV-1/MO	N/A	KY359381
RBF240	HIV-1/MO	N/A	KY359384
subtype G	CRF	Abecasis et al., 2008, J virol	N/A
CRF03_AB	CRF	Liitsola et al.. 1998, AIDS	N/A
CRF08_BC	CRF	Huang et al., 2013, J med primatol	KC914396
CRF12_BF	CRF	Carr et al.. 2001,AIDS	AF385934-6
CRF26_AU	CRF	Vidal et al.. 2009, AIDS Res Hum Retrov.	N/A
CRF31_BC	CRF	Santos et al.. 2006, AIDS	N/A
CRF32_06/A1	CRF	Adojaan et al.. 2005, J AIDS	N/A
CRF42_BF	CRF	Struck et al., 2015, AIDS Res Hum Retrov.	EU170139
CRF45_cpx	CRF	Niama et al.. 2009, AIDS Res Hum Retrov.	N/A
CRF60_BC	CRF	Simonetti et al.. 2013, Infect Genet Evol	N/A
CRF71_BF	CRF	Pessoa et al.. 2014, PLoS One	N/A
CRF87_cpx	CRF	Hu et al.. 2016, AIDS Res Hum Retrov.	N/A
CRF88_BC	CRF	Hu et al.. 2016, AIDS Res Hum Retrov.	N/A
URF_SHI	URF	Shi et al., 2004, AIDS Res Hum Retrov.	AY445524
URF_CRN	URF	Frange et al., 2008, Retrovirology	EU448296
URF_AUK	URF	Frange et al., 2008, Retrovirology	EU448295
URF_WEI	URF	Wei et al., 2013, AIDS Res Hum Retrov.	KC833436
URF_GUI	URF	Gui et al., 2013, AIDS Res Hum Retrov.	KP170487

N/A: not applicable

Table S2. Concordance of the Tat/TAR couple according to the concordance of *gag* and *nef.*

	Tat=TAR	Tat≠TAR*	Total
**discordant gag/nef**	4	4	8
**matching gag/nef**	12	7	19
**Total**	16	11	27

*or presence of a recombination breakpoint in tat or R

H0: the two variables (Tat/TAR state and gag/nef state) are independent
p-value = 0.6753 (Fisher's Exact Test for Count Data)

Table S3. Panel of reference sequences used for the recombination analyses.

HIV-1/M subtype	Genebank accesion number
A1	AF004885
A1	AM000053
A1	KT152840
A1	EU861977
A1	JX236677
A1	AF413987
A1	JX236669
B	AF042100
B	DQ007903
B	K03455
B	AB731663
B	AY423381
B	AF086817
B	EF363123
C	AF290028
C	AB485643
C	EF469243
C	KC156210
C	KC156212
C	KC156212
C	KC156220
C	AY228557
C	AB254153

HIV-1/M subtype	Genebank accesion number
D	K03454
D	M22639
D	AB485648
D	JX236668
D	AB485650
F1	FJ771008
F1	AB485658
G	AF084936
G	AB287004
CRF01	U54771
CRF01	DQ859178
CRF01	JX112869
CRF01	AB253423
CRF01	AB565504
CRF02	L39106
CRF02	AB231896
CRF02	AB049811
CRF02	AB485633
CRF02	AB485636
**HIV-1/O**	**Genebank accesion number**
O	L20587
O	L20571
O	AJ302647
P	GU111555

Supplementary Figure S1

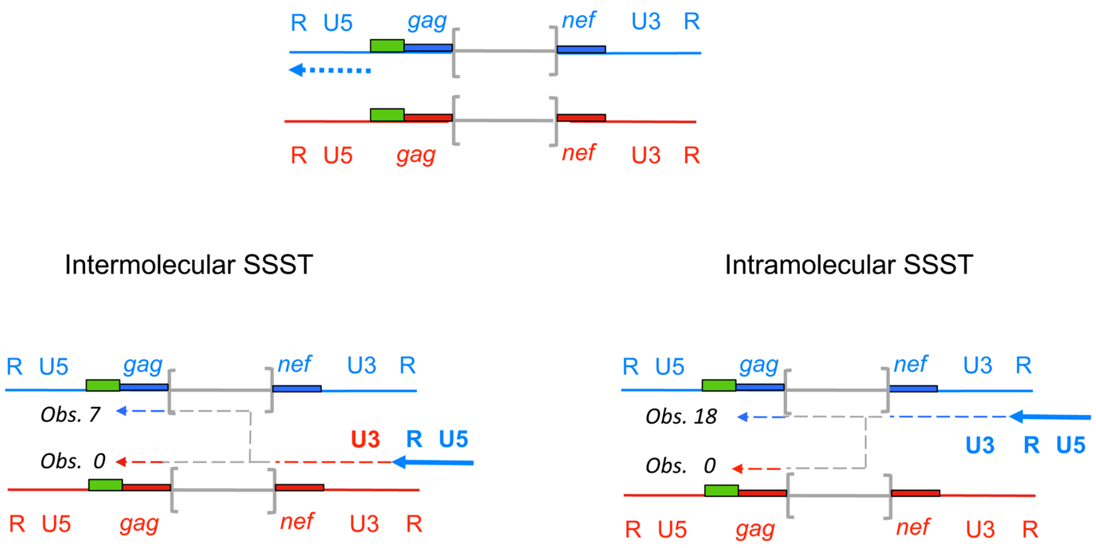

Figure S1. Pathways through which (-) DNA SSST should generate LTR with a U3/R breakpoint coupled to *discordant gag-nef* and non-recombinant LTRs coupled to *matching gag-nef*. The green rectangle represents the PBS, the boxes indicate the portions referred in the main text as *gag* and *nef* sequences for classifying proviruses into *matching gag-nef* or *discordant gag-nef*. To indicate whether they are phylogenetically matching or discordant, they are coloured in blue for the RNAs where reverse transcription is primed and in red for the other. The internal portion of the genome is drawn in grey, included in brackets. Upper drawing: synthesis of (-) strong stop DNA is indicated by a thick blue dotted arrow. Transfer of (-) strong stop DNA can then be either intermolecular or intramolecular[5-6]. In the case of intermolecular SSST, a breakpoint is generated in all cases at the U3/R junction. Depending on the number of template switching occurring during reverse transcription of internal regions, this can generate either *matching* or *discordant gag/nef* pairs. The number of cases observed in our analyses is indicated (Obs.).

Supplementary Figure S2

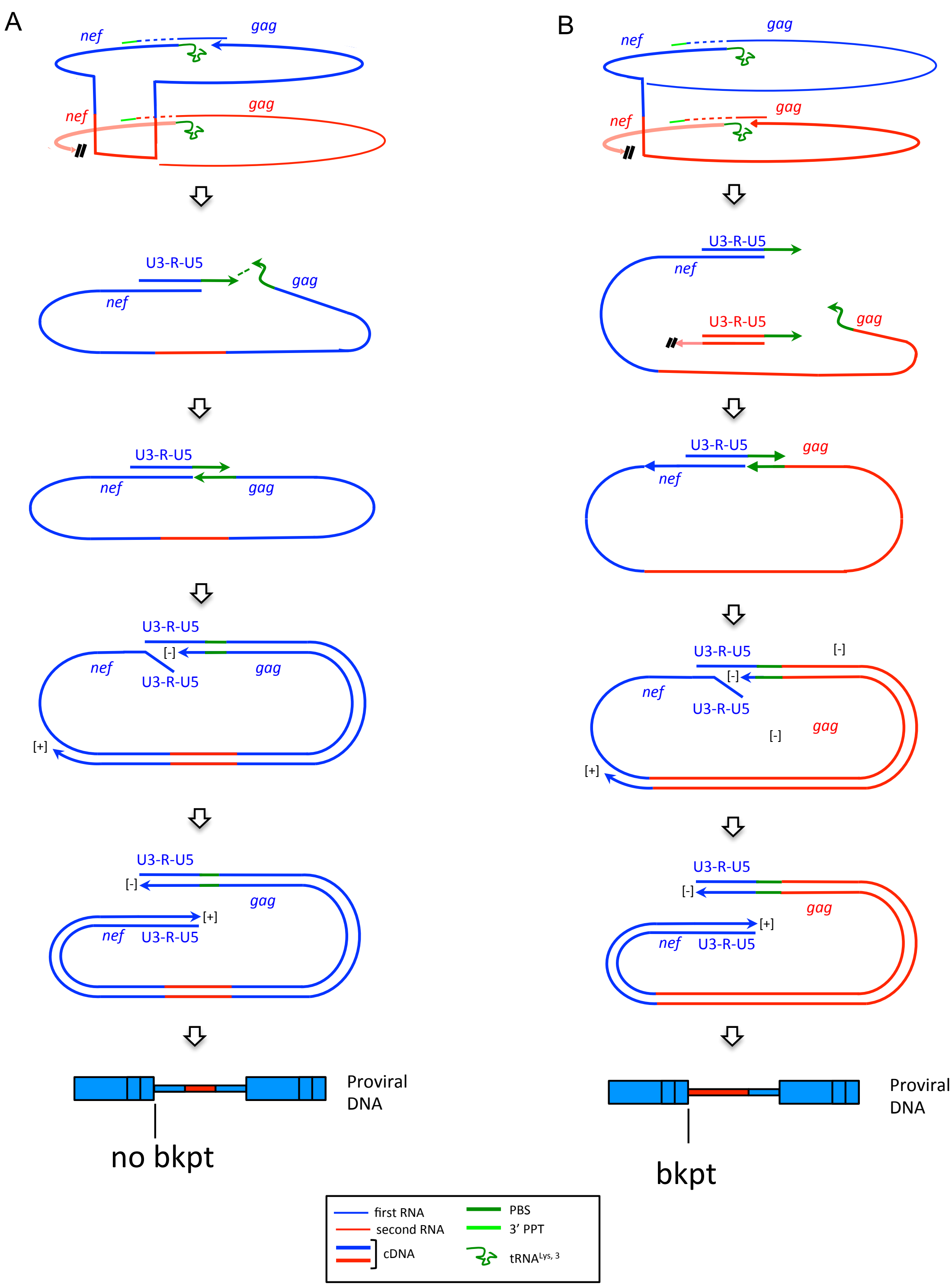

Figure S2. The description of the figure is the same as for Figure 4 of the main text. It shows that depending on the pattern of internal template switching, intramolecular (+) DNA SSST will not generate (column A) or it will generate (column B) a breakpoint at the U5/gag junction.

Supplementary sequences

Sequences of the HIV-1/MO *nef*-LTR-MA concatenated sequences.

RBF208

ATGGGAAACGCATTGAGGAGAAGCCCATTTCCAGGATGGGCAGCAATAAGAGARAGAATAAGAA
GARCTCCCCYCCCTGAGCCTGAACCATGTGCACCTGGAGTAGGACCAGTCTCCAGGGAATTAGC
AGCTAGAGGAGGGATACCCAGTTCCTTTACTCCTCAAAAYAATGCAGCCCTTGCATTCCTAGAAA
GTCACCAAGAAGAAGAAGTAGGTTTCCCAGTAAGACCTCAAGTGCCTCTAAGRCCAATGACATAT
AAACTAGCATTTGACCTCAGTTTTTAAAAGAAAAGGGGGGACTGGATGGGCTAATTTACTCCCCT
GAKAAAGCAGAGATCCTAGATCTTTGGATACATCACACTCAGGGAATCTTCCCTGATTGGCAGTG
TTACACACCAGGACCAGGAACTAGATACCCACTAACATTTGGGTGGTTATTTAAGCTAGTACCAG
TGTCAAAAGAGGAGGCAGAAGCACTAGGAAACACAAGTGAAAGGGCTAACCTACTGCATCCAGC
TTGCAGCCATGGCTTTGGAGACGCACACGGAGAGATACTGAAATGGCAGTTTGATAGATCATTAG
CCAACACCCATGTTGCTATGATAACCAACCCTGAGCTCTTCAACAAGGACTAAAACTGCTGACAT
GAAGATAGCTGACGCTGCGGGACTTTCCAACAGAGACTGCTGACACTGCGGGGACTTTCCAGTG
TGGGAGGGACAGGGGCGGTTCGGGGAGTGGCTAACCCTCAGATGCTGCATATAAGCAGCCGCT
TTCTGCTTGTACCGGGTCTCGGTTAGAGAACCAGGTCTGAGCCCGGGAGCTCCCTGGCCTCTAG
CTGAACCCGCTGCTTAACGCTCAATAAAGCTTGCCTTGAGTGAGAAGCAGTGTGTGCTCATCTGT
TCAACCCTGGTATCTAGAGATCCCTCAGATCACGTAGACTGAGGYAGAAAATCTCTAGCAGTGGC
GCCCGAACAGGGACCGGAAAGAGAAAGTGAAACCAGGGAAGAAAACCTCCGACGCAACGGGCT
CGGCTTAGCGGAGTGCACCCGCTAAGAGGCGAGAGGACTCACAGAAGGGGTGAGTAAWTTTGC
TGGCGGTGGCCAGACCTAGGGGAAGGGCGAAGTCCCTAGGGGAGGAAGATGGGTGCGAGTGC
GTCTGTGTTGAAAGGGAGCAAATTGGATGCATGGGAACAAATTAGGTTAAAGCCAGGATCTAAAA
AGAAATATCAATTAAAACATTTAGTATGGGCAAGCAGGGAGCTGGACAGATACGCATGTAATCCT
GAGCTATTAGAAACTATAGAGGGTAACGAGAAACTATTACAGCAGTTAGAGCCAGCTCTCAAGAC
AGGGTCAGACAGCCTGCAGTCACTCTGGAACACAATAGCAGTGCTCTGGTGTGTTCACAACAGA
TATARAGTTGAAGACACACAGCAGGCGATACAAAAGTTAAAGGAAGTAATGGGGAGCAGGAAGT
CTGCAGAGGTCGCTAAGGAAGACACAGGCGAAAGGCAGAGGGGTCAAAATTAC

RBF222

ATGGGTRGCAAGTGGTCAAAAGGTAGCTTTGTGGGATGGYCTCAGGTTATGGAAAGAATAAGRC
AAACTCCTCCGAGGCAAACTCCTCCAGCAGCAACAGGAGTGGGAACAGTATCTCAAGATTTAGGT
AGACATGGAGCAATCACAAGCAGTAATACAGCARCTACTAATGCTGCCTGTGCCTGGCTAGAAGC
ACAAGAAGACGAGGAGGTAGGCTTTCCAGTCAGGCCGCAGGTACCTTTGAGACCAATGACTTTT
AAGGCAGCRTTTGATCTCAGCCACTTTTTAAAAGAAAAGGGGGGACTGGATGGGTTAATTTACTC
CAAGAAAAGACAAGAGATCCTTGATCTGTGGGTCTATCACACACAAGGATTCTTCCCAGATTGGC
AGAACTACACACCAGGACCAGGGACTAGATTCCCACTGACCTTTGGGTGGTGTTTCAAGCTAGAA
CCRATGGATCCAGCAGAGGTAGAGGAAGCCAATAAAGGAGAGAACAATAGTTTATTACACCCCAT
CTGTCAGCATGGAATGGAGGACGAGGACAGAGAAGTGCTGGTCTGGAGATTTGACAGCCACCTG
GCATGGAAACACAKCGCTCGAGAGCTGCATCCGGAGTTCTACAAAGACTGCTGACACAGAAGTT
GCTGACAGGGACTTTCCGCTGGGACTTTCCGGGGGAGGTGTGGCTTGGGAGGAGTTGGGGAGT
GGCTAACCCTCAGAWGCTGCATATAAGCAGCCGCTTCTCGCCTGTACTGGGTCTCTCTTGYTAG
ACCAGATTTGAGCCTGGGAGCTCTCTGGCTAGCAGGGAACCCACTGCTTAAGCCTCAATAAATTC
AAGTAGTGTGTGCCCGTCTGTTGTGTGACTCTGGTAACTAGAGATCCCTCAGACCTTTCTAGTCT
GTGTAAAAAATCTCTAGCAGTGGCGCCCGAACAGGGACCTTTAAATWAATAGGGACTCGAAAGC
GAAAGTTCCARAGAARATCTCTCGACGCAGGGACYCGGCTGGCTGAGGTGCACGCAGCAAGAG

GCRAGAGCGGCRACTGGTGAGTACGCCAAAAAATTTGACTAGCGGAGGCTARAAGGARARARAT
GGGTGCGAGAGCGTCAGTGTTAAGTGGGGGAAAATTAGATACATGGGAAAGAATTCGCTTAAGG
CCAGGGGGAAAGAAAAAATATAAACTAAAACATATAGTATGGGCAAGCAGGGAGTTGGAAAGATT
TGCACTTAACCCTGGCCTTTTAGAATCAGCAGAAGGATGTCAACAGTTAATGGAACAGTTACAGT
CAACTCTCAAGACAGGATCAGAAGAACTTAAATCATTATTTAATACCTTAGCAGTCCTTTGGTGCG
TRCATCAAAGGATGGACATAAGAGACACCAAGGAAGCCTTAGATAAACTAGAGGAAGCGCAAAAT
AAGAGCAAGCAAAAGGAACAGCAGGCAACAGCTAGCGCAAGCTGCAGCCAAAATTAC

REC107

ATGGGGAATGCATGGAGTAAAAGTAAATTTGCAGGATGGTCAGCAGTAAGAGAGAGAATGAGGA
AAACCCCCACTAATAATCCTGAACCATGTGCACCTGGGGTAGGAGAAATCTCTAGACAGTTAGCA
GCTAGAGGAGAAATACCAGGTTCCTATACTCTTCATAACAATGCAGCCTTGGCCTTCCTAGAAGA
ACATGAAGAGGGTGAAGAAGTAGGGTTCCCAGTAACACCTCMAGTGCCTCTTAGGCCAATGACC
TATAAAGGAGCATTTGACCTCGGCTTCTTTTTAAAAGAAAAGGGAGGACTGGATGGGTTGATTTA
CTCCCATAAAAGAGCAGAGATCCTAGATCTTTGGGTGTATCRCACTCAGGGCTACTTCCCTGATT
GGCAGAACTACACACCAGGACCAGGAACCAGATACCCATTGACATTTGGATGGCCATTCAAGCTA
GTGCCAGTATCAGCAGAAGAGGCAGAAAGACTGGGCAATATGTGTGAAAGGACTACGCTCCTAC
ATCCAATCAGTAATCATGGTAGTGAAGATGAACATGGGGAAATACTGAAATGGCAGTTTGATAGTT
CAYTGGGCCGCACCCATGTTGCTTTAATAACTCACCCTCATCTCTTCAACAAGGACTAAAACTGTT
TATCTGAAGAGTGCTGACACTGCGGGACTTTCCAGCAGAGACTGCTGACGCGGCGGGGACTTTC
CAGTGTGGGAGGGATAAGGGCGGTTCGGGGAGTGGCTAACCCTCAGATGCTGCATATAAGCAG
CTGCTTCTCGCTTGTACTGGGTCTCTTTTGCTAGACCAGATTGGAGCCTGGGAGCTCTCTGGCTA
GTAGAGGAACCCACTGCTTAAGCCTCAATAAAGCTTGCCTTGAGTGCTTCAAGTAGTGTGTGCCC
ATCTGTTGTGTGACTCTGGTATCTAGAGATCCCTCAGACCACTCGAGACTGTGTAAAAATCTCTAG
CAGTGGCGCCCGAACAGGGACCGTAATAGGGACTCGAAAGCGAAAGTTCCAGAGAAGTTCTCTC
GACGCACGGACTCGGCTTGCTGAGGTGCACACAGCAAGAGGCGAGAGCGGCGACTGGTGAGTA
CGCCAATTTTTGACTAGCGGAGGCTAGAAKGAGAGAGATGGGTGCGAGAGCGTCAGTGTTAAGT
GGGGGAAAATTAGATGCGTGGGAGAAAATTCGGTTACGGCCAGGGGGAAAGAAAAAATATAGAC
TAAAACATTTAGTATGGGCAAGCAGGGAGCTGGAAAGGTTCGCACTCAACCCTGGTCTTTTAGAA
ACATCAGAAGGGTGCCAACAACTAATGGAGCAGTTACAATCAACTCTCAGAACAGGATCAGAAGA
ACTTAAGTCATTGTTTAATACAATAGCCACCCTTTGGTGTGTACATCAAAGGATAGATATTAAAGA
CACCAAGGAAGCCTTAGATAAAATAGAGGAAGTGAAAAATAAGAACAAGCAAAAGACACAGCAAG
CAGCCGCCGCAGGAAGCGGCAGCCAAAATTTC

YBF274

ATGGGTAACAAATGGTCAAAAAGTAGTATAGTGGGATGGCCTGCCATAAGGGAAAGAATAAGAAG
AACTAATCCAGCAGAAAATCCAGCAGCAGATGGGGTAGGAGCAGTATCACAAGACCTGGCAAAA
CATGGGGCAATCACAAGTAGCAATACAAAACATACTAATGCTGCCTGTGCCTGGCTAGAAGCACA
AGAAGAAGAGGGGGTGGGTTTTCCAGTCAAACCTCAGGTACCTCTAAGACCAATGACCTACAAG
GGAGCTTTAGATCTTAGCCACTTTTTAAAAGAAAAGGGGGGACTGGAAGGGCTAGTTCACTCCCA
AAGAAGACAAGAGATCCTTGATCTTTGGGTCTACCACACACAAGGCTACTTCCCTGATTGGCAGA
ACTACACACCAGGGCCAGGAACCAGATACCCACTATGCTTTGGGTGGTGCTTCGAGCTAGTACC
AGTTGATCCAAAGGTGGTAGAAGAAGAGACTGAAGGAGAGAGCAATTGTTTGCTACACCCCATAA
GCCAGCATGGAATGGATGACTCGGAGAGAGAAGTATTAGTGTGGAGATTTAACAGCAGACTAGC
ATATGAACACAAGGCCAGAGAGAAGCATCCGGAGTTCTTTAAAGACTGCTGACACCGAGTTCCTA
AAGACTGCTGATACTGAGCTTTCTACAAGGGACTTTCCGYTGGGGACTTTCCAGAGGAGGCGTG
ACTGGGGAGGGACTGGGGAGTGGCCAACCCTCAGATACTGCATATAAGCAGCCGCTTTCTGCTT
GTACCGGGTCTCGGTTAGAGAACCAGGTCTGAGCCCGGGAGCTCCCTGGCTTCTCAATAAAGCT

TGCCTTGAGTGAGAAGCAGTGTGTGCTCATCTGTTCAACCCTGGTGTCTAGAGATCCCTCAGATC
ACGTAGACTGAAGCAGAAAAATCTCTAGCAGTGGCGCCYGAACAGGGACCAGAAAGAGAAAGTG
AAACCAGGGAAGAAAACCTCCGACGCAACGGGCTCGGCTTAGCGGAGTGCACCCGCTAAGAGG
CGAGAGGAACTCACAGAAGGGTGAGTAAACTTGCTGGCGGTGCCCAGACCTAGAGGAAGGGCG
AAGTCTCTAGGGGAGGAAGATGGGTGCGAGAGCGTCTGTGTTGACAGGGAGTAAATTGRATGCA
TGGGAACAAATTAGGTTAAAGCCAGGATCTAAAAAGAAATATAGACTAAAACATTTAGTATGGGCA
AGCAGGGAGCTGGAAAGATWTGCTTGTAATCCTGAGCTACTAGAAACTGCAGAAGGCAATGAGA
AACTGWTACAACAGTTAGAACCAGCTCTCAAGACAGGGTCAGAAAGCCTRCAATCACTCTGGAA
CGCAATAGTAGTGCTCTGGTGTGTTCATAACAGATATAAAGTTGAAGATACGCAGCAGGCAATAC
AAAAGTTAAAGGAAGTAATGGGGAACAGGAAGCCYGCGGRTGYCGCTAAGGAAGACACTAGCAC
AARGCAGATGGGTCAAAACTAC

BCF212

ATGGGCAGCAAGTGGTCAAAAAGCAGCATAGTAGGATGGCCTGAGGTTAGGGAAAGAATGAGAA
GGACTCCTCCACATAATACTCCTCCAACTCCTCCAGCAGCAGTAGGAGTAGGAGCAGTATCTCAA
GATTTAGCTAGACATGGAGCAATCACAAGCAGTAATACAGCTCACACTAATGCTGACTGCGCCTG
GCTGGAAGCACAAGAAGAAGAGGAGGTAGGCTTCCCAGTCAGGCCACAGGTACCTTTAAGACCC
ATGACTTATAAAGCAGCTGTAGATCTCAGTCACTTTTTAAAAGAAAAGGGGGGACTGGATGGGTT
AATTCACTCCAGGAGAAGACAAGACATCCTTGATCTGTGGGTCTACCACACACAAGGATACTTCC
CTGATTGGCAGAATTACACGCCAGGGCCAGGGACCAGATTCCCACTAACATTTGGGTGGTGCTT
CAAGCTAGTACCAGTAGATCAAGAGGAAGTAGAGGAGGCCACTAGGGGAGAGAACAACAGCCTG
TTACATCCTATATGCCAACATGGAATGGATGATGATGAGAGAGAAGTGTTAAAGTGGCAGTTTGA
CAGCCGCCTGGCCCTAAGACACTTCGCTAGAGAAAAGCATCCGGAGTTCTACAAAGACTGCTGA
CACAGAAGTTGCTGACTGGGACTTTCCGCTGGGGACTTTCCAGGGGAGGTGTGGTTTGGGCGG
AGTTAAGGAGTGGCTAACCCTCAGATGCTGCWTATAAGCAGCCGCTTTTCGCTTGTACTGGGTCT
CTCTTGGTAGACCAGATCGAGCCTGGGAGCTCTCTGGAACTAGCGAGAGAACCCACTGCTTAAG
CCTCWATAAGCTTGCCATTGAGTGAGAAGCAGTGTGTGCTCATCTGTTCAACCCTGGTGTCTAGA
GATCCCTCAGATCACTTAGACTGAAGCAGAAAATCTCTAGCAGTGGCGCCCGAACAGGGACTTG
AAAGAGAAAGTGAAACCAGGGAAGAAATCCTCCGACGCAACGGGCTCGGCTTAGCGGAGTGCA
CCCGCTAAGAGGCGAGAGGAACTCACAGGGGTGAGTAAAATTGCTGGCGGTGGCCAGACCTAG
GGGAAGGGCGAAGTCTCTAGGGGAGGAAGATGGGTGCGAGAGCGTCTGTGTTAAGGGGAAGTA
AATTGGATGCATGGGARCAAATTAAGTTGAAGCCAGGATCTAAAAAGACATATAGACTAAAACATT
TAGTTTGGGCAAGCAGGGAGCTGGACAGATTCGCATGTAATCCTGAGCTACTAGAAAATGCAGA
GGGTAATGAGCAACTGTTACAACAGTTAGAACCAGCTCTCAAGACAGGGTCAGAAAGCCTGCAG
TCACTCTGGAACACAATAGCAGTGCTCTGGTGTGTTCACCAAAGAATTAGAGTTGGAGATACGCA
GCAGGCAGTACAAAAGTTAAGGGAGGAAATGGAGAAAAGGAAGCCTAAAAATACTGCTAAGGAA
GACACAAACTCAAGGCAGACAGGTCAAAATTAC

RBF237

ATGGGTRGCAAGTGGTCAAAAAGCAGTATAGTTGGATGGCCTGCTGTAAGGGAAAGACTAAGAC
AAACCCCTGTAGCAGCAGAKGGGGTGGGAGCAGTGTCTCAAGATTTAGATAAACATGGGGCAAT
TACAAGCAACAATACTAGGGCTACCAATGCTGACTTGGCCTGGCTGGAAGCGCARGAGGATGAG
GACGTAGGTTTYCCAGTCAGACCTCAGGTACCTTTAAGGCCAATGACTTATAAAGCAGCTGTCGA
TCTCAGCCACTTTTTAAAAGAAAAGGGGGGACTGGATGGGTTAACTTACTCCAGGAGAAGAAAAG
AAATCCTTGATTTGTGGGTCTACCACACACAAGGCTTCTTCCCTGACTGGCAGTGCTACACACCA
GGGCCAGGAACYAGGTTCCCACTGACCTTTGGATGGTGCTTCAAGCTAGTACCAGTTGATCCAG
AGGAAGTAGAAAGGGCYAATGAAGGAGAGAACAACTGTTTATTACATCCTATGAGCCTACATGGA
ATGGAGGACGACGACARGGAAGTGTTAAGATGGAAGTTTGACAGCACCCTAGCACTGAGACACT

TAGCCAAAGAGAAACATCCGGAGTACTACAAAGACTGAGAAACTGCTGACACAGAAGATCCTAAA
AGGGACTTTCCACTGGGGACTTTCCAGAAGGGCGTACCCAAGGGCGGGACCGGGGAGTGGCTA
MCCCTCAGATGCTGCATATAAGCAGCTGCTTTTCGCCTGTACTGGGTCTCTCTTGCTARACCAGA
TTTGAGCCTGGGAGCTCTCTGACTAGCAGGGAACCCACCTTGCCTTGAGTGCTTCAAGTAGTGT
GTGCCCGTCTGTTGTGTGACTCTGGTAACTAGAGATCCCTCAGACCACTCTAGATAGTGTAAAAA
TCTCTAGCAGTGGCGCCCGAACAGGGACTTGAAAGTGAAAGTTAATAGGGACTCGAAAGCGAAA
GTTCCAGAGAAGATCTCCCGACGCAGGACTCGGCTTGCTGAGGTGCACRCAGCAAGAGGCGAG
AGCGGCGACTGGTGAGTACGCCAATTTTTGACTAGCGGAGGCTAGAAGGGGAGAGATGGGTGC
GAGAGCGTCTGTRTTGTCAGGAAGCAAATTGGATGCATGGGAACAAATTAGGTTAAAGCCAGGAT
CTAAAAAGAAATATAGGCTAAAACATTTAGTATGGGCAAGCAGGGAGCTGGACAGATTCGCTTGT
AATCCTGAGCTACTAGAAACTGCAGAGGGTAATGAGAAACTGTTACAACAGTTAGAGCCAGCTCT
TAAGACAGGGTCAGAAAGCCTGCAGTCACTCTGGAACACAATAGCAGTGCTCTGGTGTGTTCACA
ATAGATTTAARGTTGAAGAYACACAGCAGGCAATACAAAAGCTAAAACAAGAAAAGGGGAAGTCT
GTGGGTACCGCTAAGGAAGACACAAGCGCAAGGCAGACAGGTCAAAATTAC

RBF240

ATGGGGAACGCATTTAAGAAAAGYAGCTTTGTRGGATGGCCAGCAGTAAGAGAAAGAATAAGAR
GAGCTCACAAGGAGCCTGAACCTGAACCATGTGCACCKGGAGTGGGAGAACTCTCCAGGGCRG
TAGCAAATAGAGGAGGGATACCAAGTTCCTTTACTCCTCAAAACAATGCAGCCCTTGCATTCCTA
GAAAGTCACCAAGAAGATGAAGAGGGAGTAGGTTTCCCAGTCAAACCTCAAGTACCTGTAAGGC
CAATGACCTATAAAGGAGCATTTGACCTCAGCTTCTTTTTAAAAGAAAAGGGAGGACTGGATGGG
TTAATTTACTCAACTAAGAGAGCAGAGATCCTGGATCTTTGGGTGCATCACACTCAGGGATTCCT
CCCWGATTGGCAGAACTACACACCAGGACCAGGAACTAGATACCCACTGACATTTGGATGGTTG
TTTAAACTAGTACCAGTGTCAGAAGCTGAGGCAGAAGACATGGGCACTGAAGGTGACAACGCTA
GTCTCCTACATCCAGCTTGTGATCATGGCTTTGAAGATCCACACGGAGAAATACTGAAATGGCAG
TTTGATAGAACATTAGGCWTCACTCATGTTGCTCAGAGAACCCACCCAGAGCTCTTCTCTAAGGA
CTAAAACTGCTAACTGAAGATTGCTGACACCGCGGGACTTTCCAGYAGAGACTGCTGACMCTGC
AGGGACTTTCCAGCGTGGGAGGGATAAAGGGCGGTCCGGGGAGTGGCTAACCCTCAGAAGCTG
CATATAAGCAGCCGCTTTCTGCTTGTACTGGGTCTCAGTTAGAGAACCAGGTTAGAGCCCGGGA
GCTCCCTRGCCTCTGGCTGAACCCACTGCTTAACGCTCAATAAACTTGCCTTRMGTGAGAAGCA
GTGTGTGCTCATCTGTTCAACCCTGGTAGCTAGAGATCCCTCAAATCACGTAAACTGAAGCAGAA
AAATCTCTAGCAGTGGCGCCCGAACAGGGACGCTGAAAGAGAAAGTGAAACCAGGGAAGAAAAC
CTCCGACGCAACGGGCTCGGCTTAGCGGAGTGCACCTGCTAAGAGGCGAGAGGAGCTCACAGA
GGGTGAGTAAATTTGCTGGCAGGTGGCCAGACCTAGGGGAAGGGCGAAGTCCCTAGGGGAGGA
AGATGGGTGCGAGAGCGTCAGTGTTAAGGGGAAGCAAATTGRATGCATGGGAACAAATTAGGTT
RAAGTCAGGATCTAAAAAGAMATATCAGCTAAAACATTTAGTATGGGCAAGCAGGRAGCTGRATA
RATTCGCATGTAATCCTGAGCTTCTAGAAACTGTAGAGGGAACTGARAAATTATTACAACAGCTAG
ARCCAGCTCTCAAGACAGGGTCAGAAAGCCTGCAGTCACTCTGGAACACTATAGCAGTGCTCTG
GTGTGTTCATAACAACTTTAGAGTAGGAGATACGCAGCAGGCAATACAAAAGTTACAAGAAGAAA
TGAAGAAAAGGAAACCTGTAGATACTGCTAAGGAAAACTCAAGGCAGACAGGTCA
